# Information transfer of an Ising model on a brain network

**DOI:** 10.1186/1471-2202-14-S1-P376

**Published:** 2013-07-08

**Authors:** D Marinazzo, M Pellicoro, Guo-Rong Wu, L Angelini, JM Cortes, S Stramaglia

**Affiliations:** 1 Faculty of Psych. and Ed. Sciences, Dept. of Data Analysis, Ghent University, 1, B-9000 Ghent, Belgium; 2 Dipartimento di Fisica e Istituto Nazionale di Fisica Nucleare, Sezione di Bari, Bari, 70126, Italy; 3 Key Lab. for NeuroInformation, School of Life Science and Technology, UESTC, Chengdu, 610054, China; 4 Biocruces Health Research Institute, Barakaldo, Spain

## 

We consider an anatomical connectivity matrix (66 nodes), obtained via diffusion spectrum imaging (DSI) and white matter tractography [1], describing the brain at a coarse scale, and implement on it an Ising model with Glauber dynamics, estimating the transfer of information between spins. Tuning the temperature to criticality, so as to render the system characterized by long range correlations, we find that the critical state is characterized by the maximal amount of total information transfer among variables and exhibits signature of the law of diminishing marginal returns, a fundamental principle of economics which states that when the amount of a variable resource is increased, while other resources are kept fixed, the resulting change in the output will eventually diminish [2]. The origin of such behavior resides in the structural constraint related to the fact that each node of the network may handle a limited amount of information. The modulation of this phenomenon is analyzed evaluating, at each node, the ratio R between the outgoing and the incoming information. It turns out that R is related to (but not fully explained by) the in-strength of the brain network: nodes with high R are those more prone to became bottlenecks as the information transfer increases. The regions which are recognized as potential bottlenecks by the present analysis are symmetrical in the two hemisphere, and are Superior Frontal Cortex, Precuneus, Superior Temporal Cortex, Medial and Lateral Orbitofrontal Cortex. Some of these regions are considered as hubs both for the structural and for the functional connectome. Furthermore the temperature of the Ising model has an influences on the ratio between intra-hemispheric and inter-hemispheric information transfer, which grows with the temperature, though not uniformly.

## Conclusions

Criticality has been proposed to characterize brain signals [3]. The critical state of an Ising model on a simulated brain network is characterized by having the maximal amount of total flow of information, with some units being close to be receiving the maximal amount of input information.

Recent works have simulated brain activity implementing several dynamical on the connectome structure, retrieving in some cases correlation-based networks similar to those observed from the analysis of neuroimaging data (mainly fMRI at rest). The present work extends the analysis to dynamical networks who take into account lagged and directional influences, concentrating for this abstract on the nodes that more prominently express this disparity between incoming and outgoing information.

**Figure 1:**
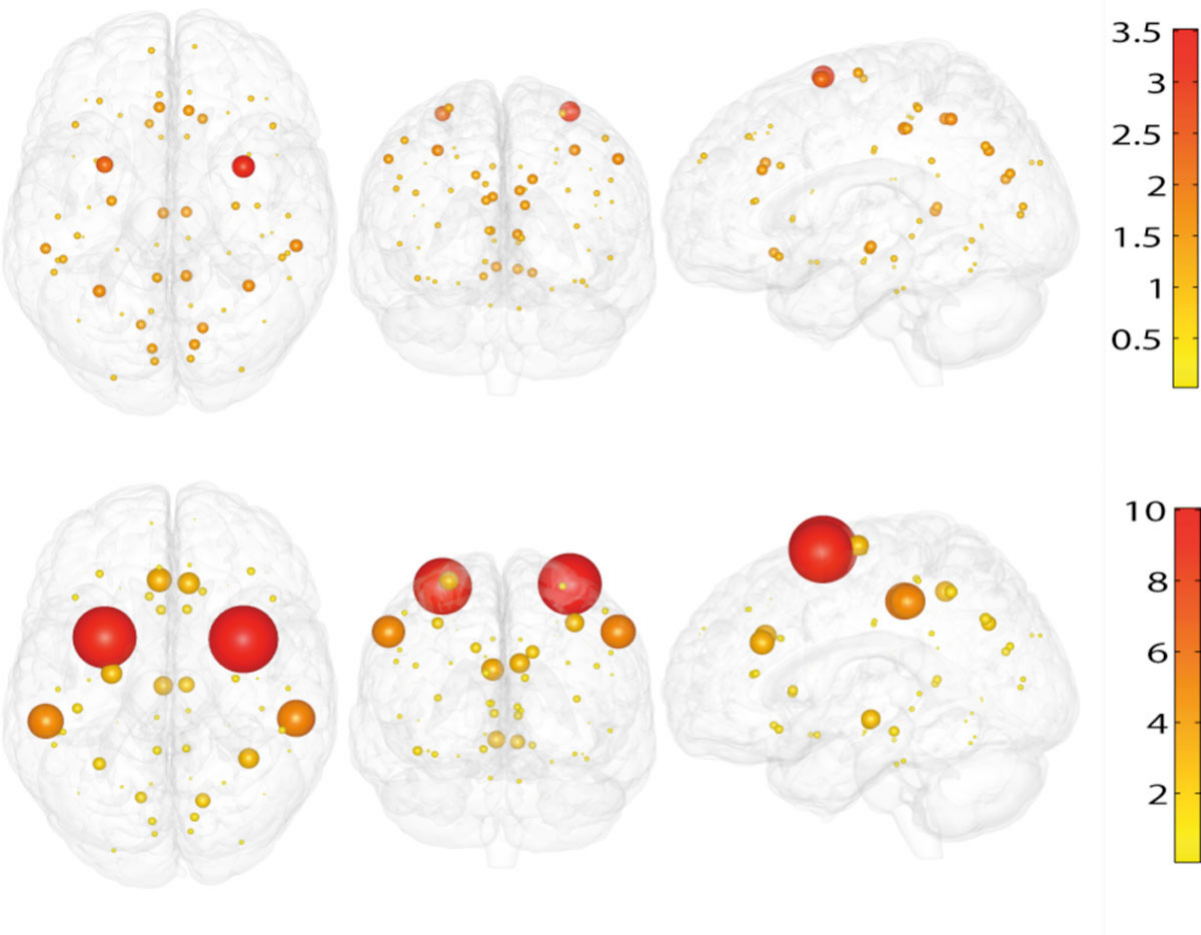
**The value of R for the 66 regions for the linear model with threshold (top) and for the Ising model (bottom)**. The size of the spheres is proportional to the value of R.
